# The Efficacy and Safety of Propiverine Hydrochloride in Patients with Overactive Bladder Symptoms Who Poorly Responded to Previous Anticholinergic Agents

**DOI:** 10.1155/2011/714978

**Published:** 2011-06-28

**Authors:** Naoya Masumori, Shintaro Miyamoto, Taiji Tsukamoto, Seiji Furuya, Akihiko Iwasawa, Takashi Sato, Naoki Itoh, Akihiko Shibuya, Toshiro Oda

**Affiliations:** ^1^Department of Urology, Sapporo Medical University School of Medicine, Sapporo 060-8543, Japan; ^2^Furuya Hospital, Urology service, Kitami 090-0065, Japan; ^3^Iwasawa Clinic, Urology service, Sapporo 060-0061, Japan; ^4^Nisshin Urological Clinic, Urology service, Tomakomai 053-0833, Japan; ^5^NTT East Japan Sapporo Hospital, Division of Urology, Sapporo 060-0061, Japan; ^6^Kaguraoka Urological Clinic, Urology service, Asahikawa 078-8315, Japan; ^7^Kiyota Urological Clinic, Urology service, Sapporo 004-0841, Japan

## Abstract

*Objectives*. To prospectively examine the efficacy and safety of propiverine hydrochloride in patients with overactive bladder (OAB) symptoms who poorly responded to previous treatment with solifenacin, tolterodine or imidafenacin. *Methods*. Patients aged ≥20 with persisting OAB symptoms (≥6 in OAB symptom score (OABSS)) even after at least 4-week treatment using solifenacin, tolterodine or imidafenacin were enrolled. Propiverine 20 mg/day was administered for 12 weeks to 70 patients who desired the further improvement of OAB symptoms and 3 who had intolerable adverse events of previous drugs. The OABSS and postvoid residual urine volume (PVR) were determined before and at 4 and 12 weeks of treatment. *Results*. Of 73 patients enrolled (29 males and 44 females, median age 71 years), 52 completed the protocol treatment. The OABSS was significantly improved by propiverine treatment (9.0 at baseline, 6.2 at 4 weeks, 6.3 at 12 weeks (*P* < 0.001)). The scores of OAB symptoms (nighttime frequency, urgency and urge incontinence) except daytime frequency also improved significantly. No increase in PVR was observed. The most frequent adverse event was dry mouth (13.7%), followed by constipation (6.8%). *Conclusions*. Propiverine is useful to improve OAB for patients who poorly respond to solifenacin, tolterodine or imidafenacin.

## 1. Introduction

Overactive bladder (OAB), a syndrome that presents urinary urgency as an essential symptom [1], has a great impact on the quality of life (QOL) of the patient. A nationwide survey conducted in Japan [2] demonstrated that the estimated prevalence of OAB was 12.4% in the general population over age 40. The study showed that 11.2% and 53.0% of subjects with OAB reported “an impact” and “a slight impact” on their QOL by OAB symptoms, respectively, through the impairment of mental health, vitality, physical activity, domestic work, and business work. Since the actual number of patients with OAB has been increasing in parallel with the advancement of aging society, the appropriate treatment strategy should be established immediately.

Drugs with anticholinergic activity are the first-line drugs to treat OAB symptoms [3]. Propiverine hydrochloride is an agent with calcium antagonistic activity in addition to anticholinergic activity [4]. A double-blind randomized control study demonstrated that propiverine was superior to oxybutynin hydrochloride in terms of the improvement of pollakisuria and urinary incontinence associated with neurogenic bladder and unstable bladder [5]. Since its launch in 1993, propiverine hydrochloride has been widely used in clinical settings in Japan. On the other hand, three new anticholinergic agents, solifenacin succinate, tolterodine tartrate, and imidafenacin, were successively approved and marketed in recent years. Different from propiverine, these drugs have no calcium antagonistic activity. Differences in clinical efficacy remain unknown between propiverine with calcium antagonistic activity and new anticholinergic agents without the activity. In the present study, we examined the clinical efficacy and safety of propiverine for patients with OAB symptoms who poorly responded to previous treatment with solifenacin, tolterodine, or imidafenacin.

## 2. Methods

The present study was conducted in patients aged ≥20 who had ≥6 points in overactive bladder symptom score (OABSS) [6] even after at least 4-week treatment with solifenacin, tolterodine, or imidafenacin. Of them, 70 patients who desired the further improvement of OAB symptoms and 3 patients who desired to change previous anticholinergic drugs because of their adverse events were enrolled in the study. Propiverine 20 mg q.d. was administered for 12 weeks. Subjective symptoms and objective findings were assessed by the OABSS and determination of PVR, respectively, before and at 4 and 12 weeks of treatment. Information of adverse events reported during the treatment period was collected.

Paired *t*-test was performed to examine changes in the OBASS and PVR. A combined analysis model was used to investigate differences in therapeutic effects by previous anticholinergic agent and by gender.

The present study was approved by the institutional review board at Sapporo Medical University (no. 19–48) and performed at 19 institutions as a multi-institutional study between January 2008 and December 2009. Written informed consent was obtained from all participants.

## 3. Results

Twenty-nine men and 44 women were enrolled in the study. Median age of 73 patients was 71 years. No difference was observed in age (mean ± standard deviation) between men (72.6 ± 11.2 years) and women (70.2 ± 11.4 years, *P* = 0.38). Regarding previous anticholinergic agents, 34, 14, and 25 patients received a standard dose of solifenacin, tolterodine, and imidafenacin, respectively. These agents were given for <8 weeks in 22 (30.1%), 8–12 weeks in 11 (15.1%), and ≥12 weeks in 40 (54.8%) patients. Of the 29 men, 15 (51.2%) had received an *α*1-adrenoceptor antagonist at the entry. The drug was continued without change in the dosage during the study period.

Of 73 subjects, 52 (71.2%) completed the protocol treatment and 21 (28.8%) withdrew from the study. The main reasons of discontinuation were “no visit to the hospital” in 9 and “adverse events” in 7, followed by “withdrawal of the consent” in 2 and deterioration of comorbid diseases in 2.

Of 52 subjects who completed the protocol treatment, the OABSS improved significantly at 4 and 12 weeks of propiverine treatment (Table 1). Among OAB symptoms, nighttime frequency, urgency, and urgency incontinence except daytime frequency showed significant improvements by propiverine treatment. The efficacy of propiverine was analyzed according to previous anticholinergic agent and to gender (Table 2). Regardless of the type of previous anticholinergic agents, the OABSS improved significantly. No significant difference in OABSS change was observed among the previous drugs (*P* = 0.31). Although the OABSS was significantly improved by propiverine treatment in both men and women, propiverine was less effective in men than in women (*P* < 0.01).

The effects of propiverine on PVR were analyzed in 50 subjects who completed the protocol treatment and whose PVR was measured. PVRs (mean ± standard deviation) were 31 ± 41, 32 ± 43 (*P* = 0.76), and 32 ± 42 mL (*P* = 0.81) before and at 4 and 12 weeks of treatment, respectively. PVRs did not increase significantly both in men (*n* = 19) and women (*n* = 31) at 4 and 12 weeks of propiverine treatment.

The overall incidence of adverse events was 21.9% (16/73), and all were mild and moderate in intensity (Table 3). Dry mouth was the most frequently reported adverse event in 13.7% (10/73), followed by constipation in 6.8% (5/73). Seven patients discontinued the protocol treatment due to adverse events (dry mouth in 4, urinary retention in 1, gastric distress in 1, and anorexia in 1). Urinary retention was developed in a 70-year-old woman 78 days after administration. All adverse events except anorexia were recovered by discontinuation of the drug.

## 4. Discussion

Propiverine hydrochloride inhibits abnormal contractions of bladder smooth muscle* in vivo* through not only its anticholinergic activity but also its concurrent calcium antagonistic activity [7]. Calcium antagonistic activity has not been reported with other anticholinergic agents such as solifenacin, tolterodine, and imidafenacin. Although both cholinergic and atropine-resistant contractions are involved in the development of unstable bladder [8], elderly patients with OAB are likely to show the increased involvement of atropine-resistant contractions that poorly respond to anticholinergic agents. Propiverine that significantly inhibits atropine-resistant contractions through the calcium antagonistic activity [9] may be useful for patients with OAB who show the increased involvement of atropine-resistant contractions.

In real-world clinical practice, an anticholinergic agent with insufficient efficacy is often switched to another anticholinergic agent. However, this modality has not been clearly proven yet. In the present study, propiverine significantly improved the OABSS in poor responders to previous treatment with solifenacin, tolterodine, or imidafenacin. We speculate that the calcium antagonistic activity which is specific to propiverine allowed the drug to exert efficacy also in patients who had shown the insufficient efficacy of other anticholinergic agents without the activity.

 In the present study, a significant gender difference was found in the efficacy of propiverine. Although the reasons why the efficacy of propiverine appeared more rapidly in women than in men remain unknown, differences in the etiology of OAB or pharmacokinetics/pharmacodynamics between men and women may be involved. This is a topic to be investigated in the future because gender difference has never been studied.

Anticholinergic agents are known to develop systemic adverse reactions such as dry mouth, constipation, and impairment of eye accommodation. Propiverine is a drug that is nonselective to muscarinic receptor subtypes. In a treatment outcome survey conducted in approximately 10,000 patients in Japan, the safety of propiverine was verified [10]. Although the incidences of dry mouth and constipation in the present study were 13.7% and 6.8%, respectively, the values were equivalent to those in previous studies.

In the present study, 9 (42.9%) of 21 patients who discontinued the protocol treatment never visited the hospital. The previous survey [10] demonstrated that the major reasons for treatment discontinuation were “symptom improvement” and “no visit to the hospital.” Tanaka and Masumori [11] also reported that the improvement of symptoms was a major reason of termination in 21 (68%) of 31 patients who discontinued propiverine treatment. Thus, OAB symptoms were likely to be improved by the short-term intake of propiverine in some patients, which made them never come back to the hospital. 

Since this study did not conduct as a double-blind placebo-controlled trial, the placebo effect may be involved in symptomatic improvement. In addition, the improvement may be obtained even by switching among solifenacin, tolterodine, and imidafenacin [12–14]. However, to date, no efficient therapeutic strategy has been established in patients who poorly responded to treatment with anticholinergic agents, because of the lack of well-designed placebo-controlled trials. The observation that propiverine was effective for poor responders to other anticholinergic agents is of great clinical relevance. Therefore, propiverine is considered as one of options for a second-line treatment even if the efficacy of solifenacin, tolterodine, or imidafenacin is insufficient, although the placebo-controlled trial is definitely necessary to prove it.

## Figures and Tables

**Table 1:** Changes in overactive bladder symptom scores in 52 patients treated with propiverine hydrochloride.

	Baseline	4 weeks	12 weeks
OABSS	9.0 ± 2.2^(1)^	6.2 ± 3.3**	6.3 ± 3.3**
Daytime frequency	0.9 ± 0.4	0.8 ± 0.6	0.8 ± 0.4
Nighttime frequency	2.3 ± 0.8	1.9 ± 0.8*	1.9 ± 0.9**
Urgency	3.7 ± 0.9	2.3 ± 1.7**	2.2 ± 1.6**
Urgency incontinence	2.2 ± 1.6	1.2 ± 1.6**	1.4 ± 1.6*

OABSS: overactive bladder symptom score.

^(1)^Mean ± standard deviation.

*P < 0.01, **P < 0.001 (versus baseline, paired *t*-test).

**Table 2:** Changes in overactive bladder symptoms by previous anticholinergic agent and by gender.

Variables	Overactive bladder symptom scores			
	Baseline	4 weeks	12 weeks	*P*-value^(2)^
Solifenacin (*n* = 27)	9.4 ± 2.5^(1)^	7.2 ± 3.9**	6.5 ± 3.9**	*P* = 0.31
Tolterodine (*n* = 10)	8.4 ± 1.4	4.7 ± 1.7**	5.9 ± 2.5*	
Imidafenacin (*n* = 15)	8.7 ± 2.1	5.4 ± 2.2**	6.1 ± 2.8**	

Male (*n* = 21)	8.5 ± 1.9	6.9 ± 3.2**	6.4 ± 2.9**	*P* < 0.01
Female (*n* = 31)	9.4 ± 2.4	5.7 ± 3.3**	6.1 ± 3.6**	

^(1)^Mean ± standard deviation.

^(2)^Comparison among groups (combined analysis model).

*P < 0.01, **P < 0.001 (versus baseline, paired *t*-test).

**Table 3:** Adverse events in 73 patients.

Adverse events	*n* (%)	Mild	Moderate	Severe	Serious
Dry mouth	10 (13.7)	6	4	0	0
Constipation	5 (6.8)	5	0	0	0
Urinary retention	1 (1.4%)	0	1	0	0
Blurred vision	1 (1.4%)	1	0	0	0
Urinary tract infection	1 (1.4%)	1	0	0	0
Gastric distress	1 (1.4%)	0	1	0	0
Anorexia	1 (1.4%)	0	1	0	0

Mild: no impairment of activity of daily life.

Moderate: limited activity of daily life.

Serve: impossible activity of daily life.

Serious: life-threatening.

## References

[1] Abrams P, Cardozo L, Fall M (2002). The standardisation of terminology of lower urinary tract function: report from the standardisation sub-committee of the international continence society. *Neurourology and Urodynamics*.

[2] Homma Y, Yamaguchi O, Hayashi K (2005). An epidemiological survey of overactive bladder symptoms in Japan. *British Journal of Urology International*.

[3] Yamaguchi O, Nishizawa O, Takeda M (2009). Clinical guidelines for overactive bladder. *International Journal of Urology*.

[4] Haruno A, Yamasaki Y, Miyoshi K (1989). Effects of propiverine and its metabolites on isolated guinea pig urinary bladder. *Folia Pharmacol Japan*.

[5] Stohrer M, Murtz G, Kramer G, Schnabel F, Arnold EP, Wyndaele JJ (2007). Propiverine compared to oxybutynin in neurogenic detrusor overactivity: results of a randomized, double-blind, multicenter clinical study. *European Urology*.

[6] Homma Y, Yoshida M, Seki N (2006). Symptom assessment tool for overactive bladder syndrome: overactive bladder symptom score. *Urology*.

[7] Yamada A, Kobayashi Y, Aita T (2008). Effect of propiverine on the atropine-resistant contraction of urinary bladders in aged rats. *Yamagata Medical Journal*.

[8] Anderson KE (1993). Pharmacology of lower urinary tract smooth muscles and penile erectile tissues. *Pharmacological Reviews*.

[9] Sugiyama Y, Yoshida M, Masunaga K (2008). Pharmacological effects of propiverine and its active metabolite, M-1, on isolated human urinary bladder smooth muscle, and on bladder contraction in rats. *International Journal of Urology*.

[10] Itoh K, Nishi T (2002). Post marketing surveillance of propiverine hydrochloride (BUP-4 tablets) in pollakiuria and urinary incontinence. *Japanese Pharmacology and Therapeutics*.

[11] Tanaka Y, Masumori N (2008). Clinical follow-up of patients treated with propiverine hydrochloride for pollakisuria and/or urinary incontinence. *Japanese Journal of Urological Surgery*.

[12] Chancellor MB, Zinner N, Whitmore K (2008). Efficacy of solifenacin in patients previously treated with tolterodine extended release 4 mg: results of a 12-week, multicenter, open-label, flexible-dose study. *Clinical Therapeutics*.

[13] Swift SE, Siami P, Forero-Schwanhaeuser S (2009). Diary and patient-reported outcomes in patients with severe overactive bladder switching from tolterodine extended release 4 mg/day to solifenacin treatment: an open-label, flexible-dosing, multicentre study. *Clinical Drug Investigation*.

[14] Onishi T, Kato M, Hoshina A (2010). The efficacy and safety for switchover treatment with second generation anti-muscarinic agent therapy in patients with overactive bladder. *J New Rem Clin*.

